# Correction to: Phage-centric ecological interactions in aquatic ecosystems revealed through ultra-deep metagenomics

**DOI:** 10.1186/s40168-020-00828-7

**Published:** 2020-03-19

**Authors:** Vinicius S. Kavagutti, Adrian-Ştefan Andrei, Maliheh Mehrshad, Michaela M. Salcher, Rohit Ghai

**Affiliations:** 1 Department of Aquatic Microbial Ecology, Institute of Hydrobiology, Biology Centre of the Academy of Sciences of the Czech Republic, Na Sádkách 7, 370 05 České Budějovice, Czech Republic; 2 Department of Ecosystem Biology, Faculty of Science, University of South Bohemia, Branišovská 1760, 370 05 České Budějovice, Czech Republic; 3 Limnological Station, Institute of Plant and Microbial Biology, University of Zurich, Seestrasse 187, 8802 Kilchberg, Switzerland

**Correction to: Microbiome**

**https://doi.org/10.1186/s40168-019-0752-0**

Following publication of the original article [1], the authors reported that an affiliation of the first author was missing. The first author Vinicius S. Kavagutti is also affiliated to Department of Ecosystem Biology, Faculty of Science, University of South Bohemia, Branišovská 1760, 370 05, České Budějovice, Czech Republic. The changes are reflected in this article.
