# Supplementary material for: Phage-centric ecological interactions in aquatic ecosystems revealed through ultra-deep metagenomics
Source: Microbiome. 2019 Oct 20;7:135. doi: 10.1186/s40168-019-0752-0 (PMC6802176; doi:10.1186/s40168-019-0752-0)
Supplement: Supplementary file 1 — Additional file 1: Figure S1. a) Water temperature along a depth profile from June 2015 to December 2017 in Římov reservoir. b) Oxygen concentrations along a depth profile from March 2015 to November 2017 in Římov reservoir. Red circles indicate the time and depth of the samples. Oxygen measurements were not available for all time points. Figure S2. 16S rRNA-based abundances of prokaryotic groups. Figure S3. Length distribution of Viral RefSeq phage genomes (inset: enlarged view of phages up to length 100 K). Figure S4. Comparative GC% distributions of freshwater metagenomic data for Římov epilimnion (n = 10 datasets), Římov hypolimnion (n = 8 datasets) and Jiřická (n = 5 datasets). Figure S5. Phage proteomic tree showing relationships of freshwater (n = 1330), marine (n = 1202) and RefSeq (n = 1887) phages used in this study. Figure S6. Genomic variations in a persistent actinophage (Cluster1). Figure S7. Relative abundance of 279 Jiřická phages in 5 metagenomes (coverage per Gb of metagenome normalized by Z-score). [file 40168_2019_752_MOESM1_ESM.pdf]

Supplementary Information

Phage-centric ecological interactions in aquatic ecosystems revealed through ultra-deep metagenomics

Vinicius S. Kavagutti[1], Adrian-Ştefan Andrei[1], Maliheh Mehrshad[1], Michaela M. Salcher[1,2], Rohit Ghai[1*]

[1]*Department of Aquatic Microbial Ecology, Institute of Hydrobiology, Biology Centre of the Academy of Sciences of the Czech Republic, České Budějovice, Czech Republic*.

[2]*Limnological Station, Institute of Plant and Microbial Biology, University of Zurich, Seestrasse 187, 8802 Kilchberg, Switzerland*.

*Corresponding author: Rohit Ghai

Department of Aquatic Microbial Ecology, Institute of Hydrobiology, Biology Centre of the Academy of Sciences, Na Sádkách 7, 370 05, České Budějovice, Czech Republic

Phone: +420 387 775 881

Fax: +420 385 310 248

E-mail: ghai.rohit@gmail.com

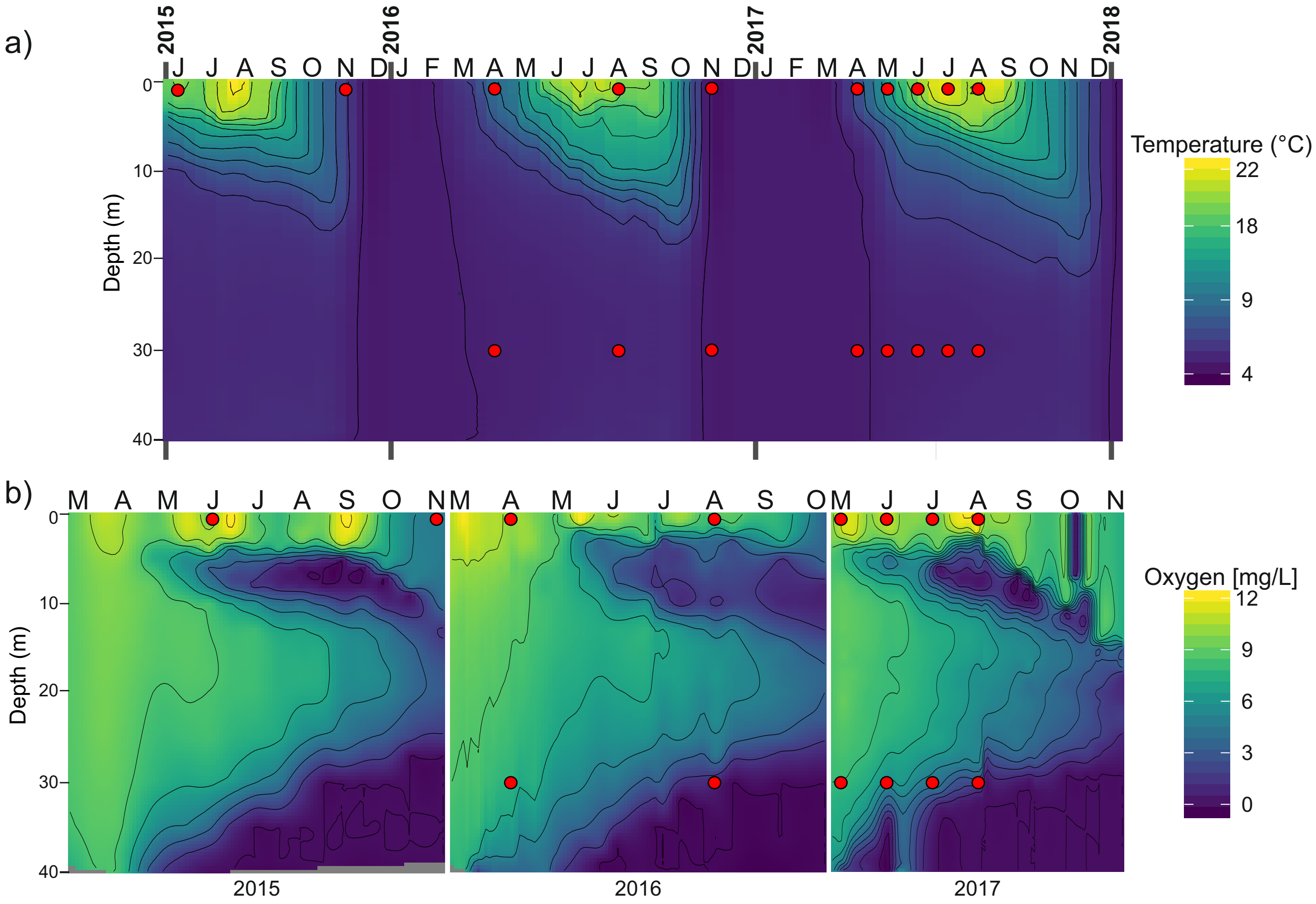

Supplementary Figure S1. a) Water temperature along a depth profile from June 2015 to December 2017 in Římov reservoir. b) Oxygen concentrations along a depth profile from March 2015 to November 2017 in Římov reservoir. Red circles indicate the time and depth of the samples. Oxygen measurements were not available for all time points.

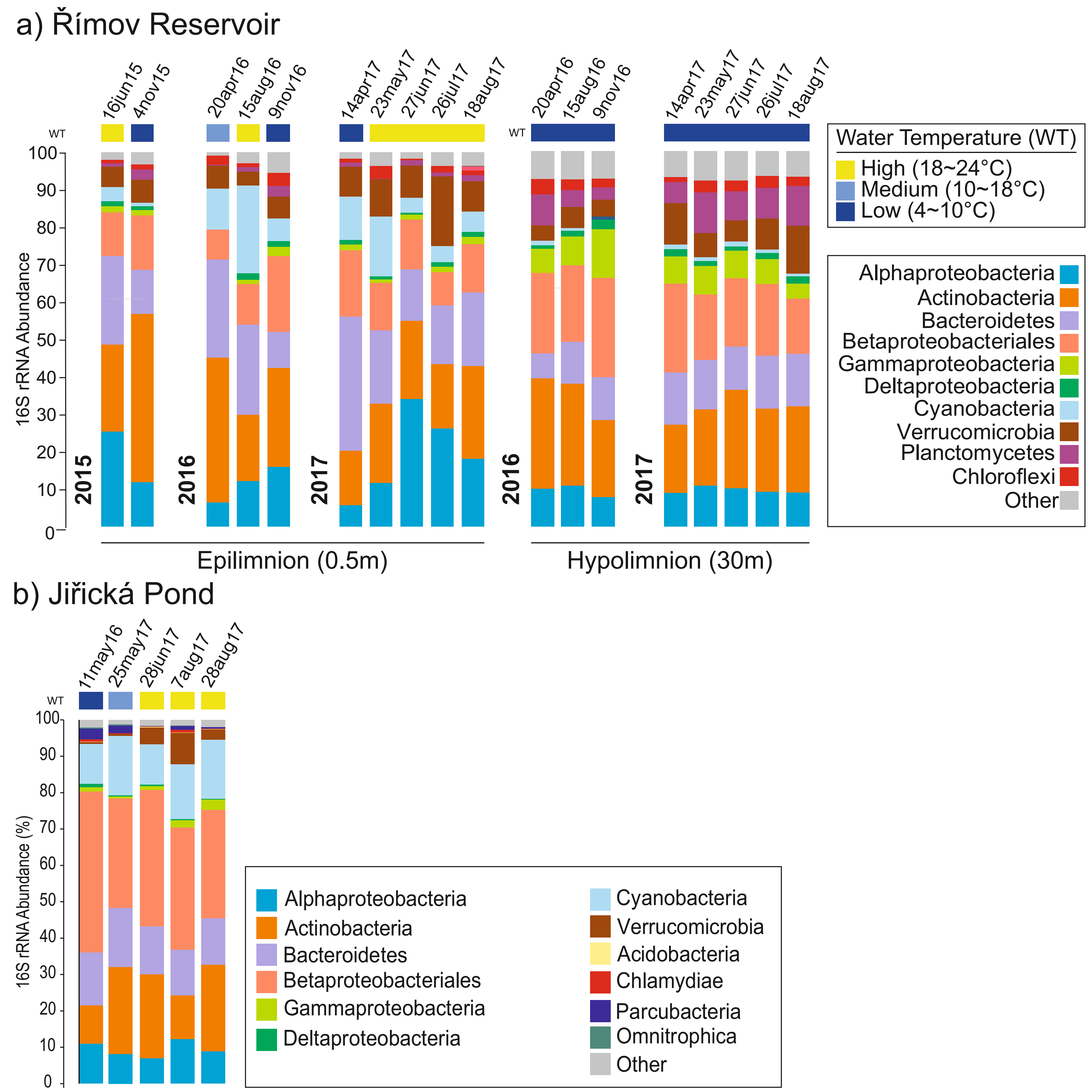

Supplementary Figure S2. 16S rRNA based abundances of prokaryotic groups. The figure depicts the SILVA SSU (Ref NR 99 132) classification of 16S rRNA reads in the metagenomes of (a) Římov reservoir and (b) Jiřická Pond. Water temperature at the time of sampling is also indicated (scale top right).

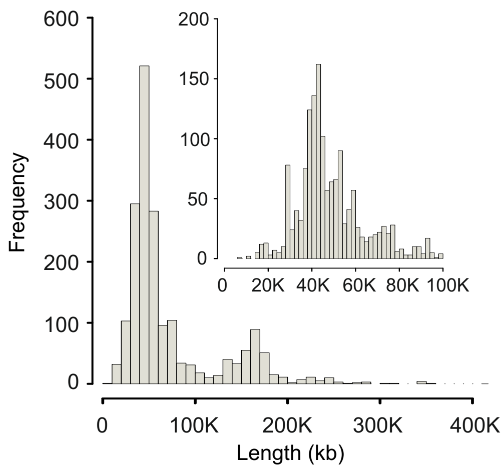

Supplementary Figure S3. Length distribution of Viral RefSeq phage genomes (inset: enlarged view of phages up to length 100K).

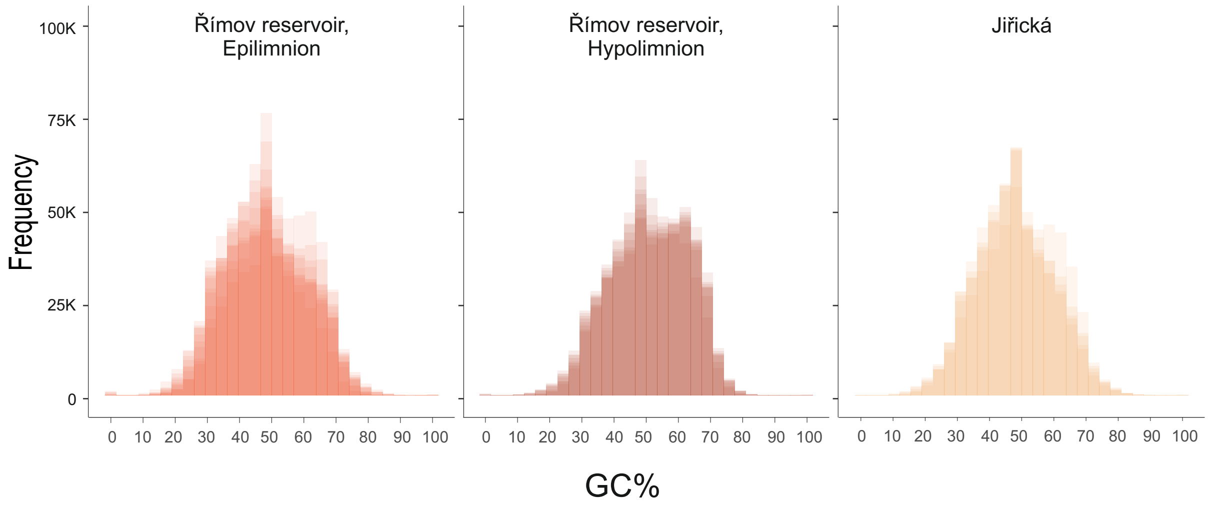

Supplementary Figure S4. Comparative GC% distributions of freshwater metagenomic data for Římov epilimnion (n=10 datasets), Římov hypolimnion (n=8 datasets) and Jiřická (n=5 datasets).

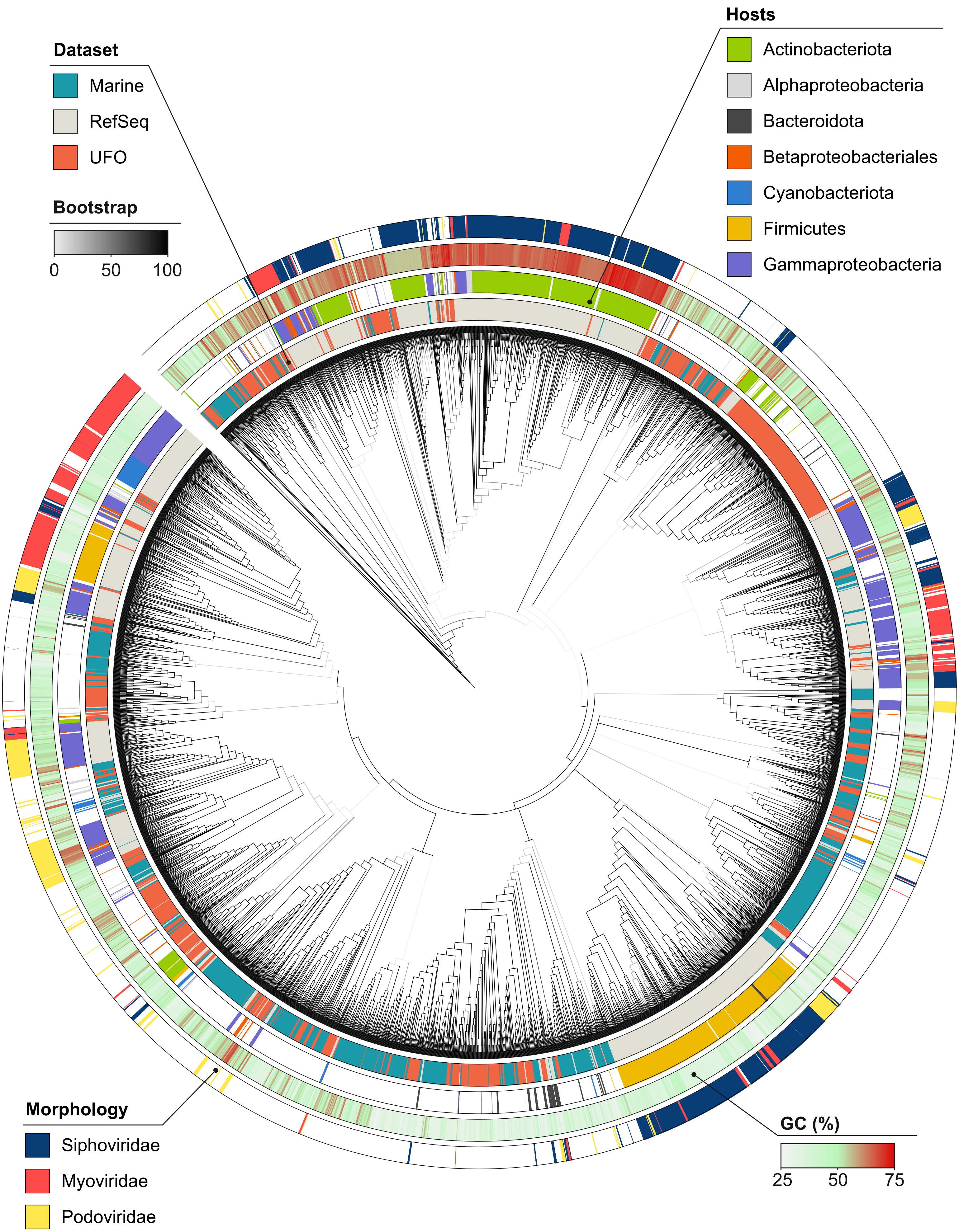

Supplementary Figure S5. Phage proteomic tree showing relationships of freshwater (n=1330), marine (n=1202) and RefSeq (n=1887) phages used in this study. For more details see methods. From inside out: Gray colored tree branches represent bootstrap values below 100, phage dataset, predicted hosts, genome GC% and known phage morphology (for RefSeq). Figure made with iTOL (http://embl.itol.de).

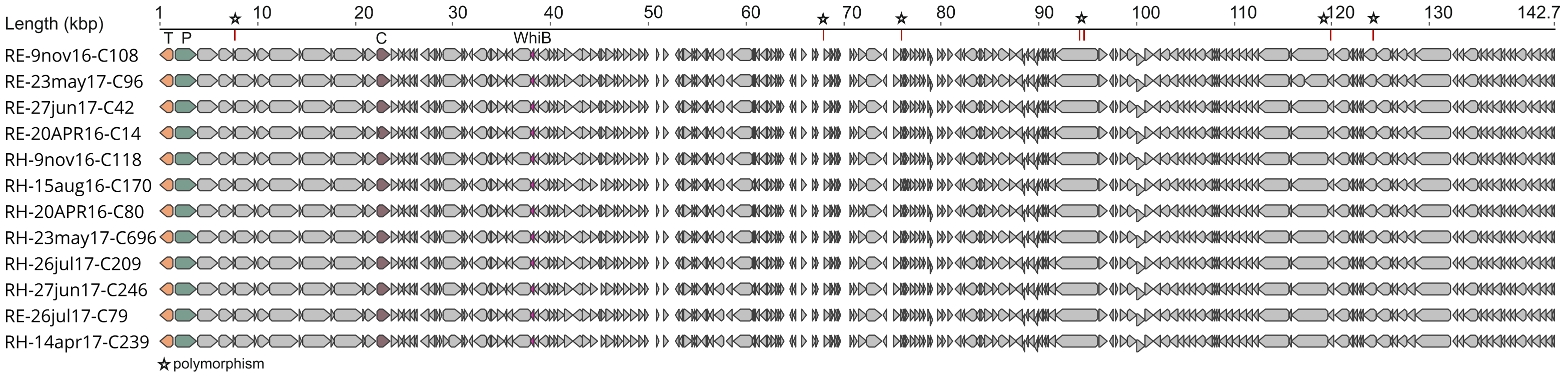

Supplementary Figure S6. Genomic variations in a persistent actinophage (Cluster1). Whole genome alignment of a phage genome that was recovered independently 12 times. The variant polymorphisms are indicated with a "*" at the top. T: Terminase Large Subunit, C: Major Capsid Protein, P: Portal protein.

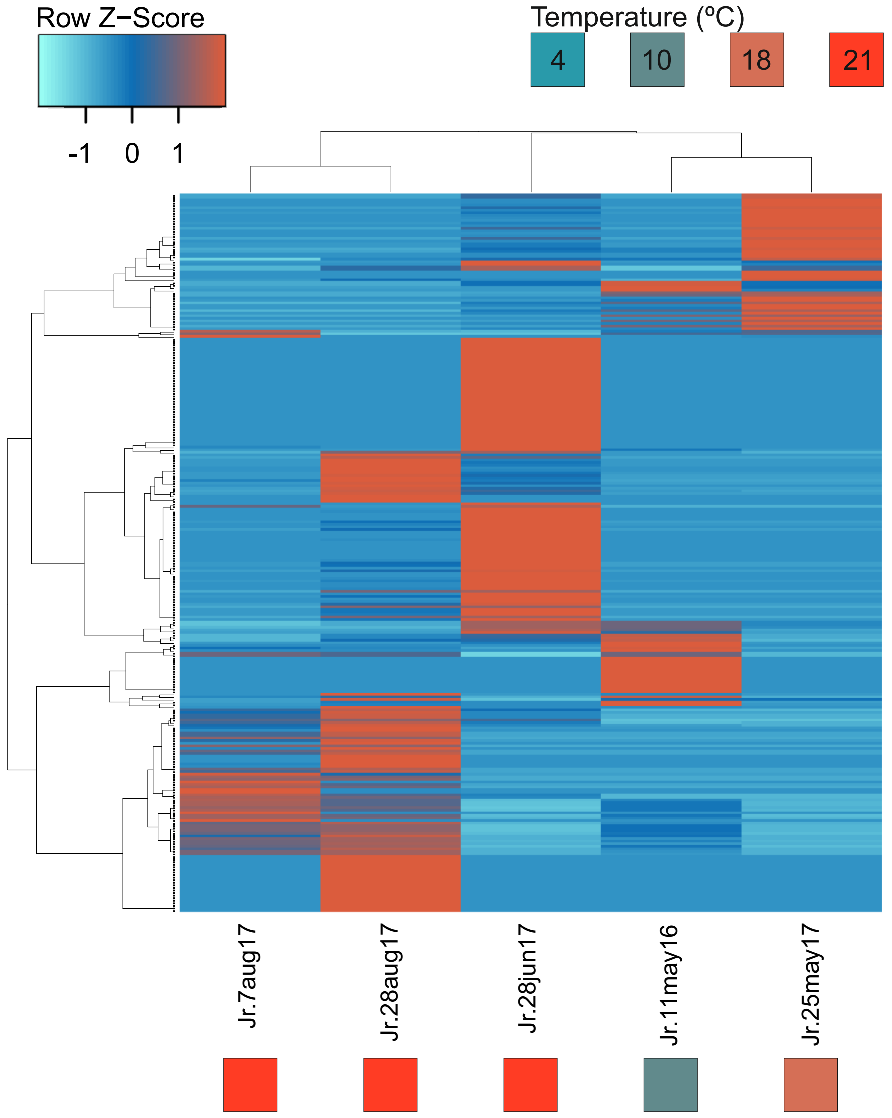

Supplementary Figure S7. Relative abundance of 279 Jiřická phages in 5 metagenomes (coverage per Gb of metagenome normalized by Z-score). Phages are clustered by sample and abundance by average linkage (with Spearman Rank correlation method). Columns are annotated with the temperature and the sampling date. Temperature color key is shown at top right.

List of Supplementary Tables

Supplementary Table S1: Metagenomic datasets used in this study

Supplementary Table S2: Freshwater phage genome information

Supplementary Table S3: Phage Genomes encoding ADP-ribosyltransferase toxin (pfam domain PF03496)

Supplementary Table S4: Freshwater phage genomes encoding ribosomal proteins

Supplementary Table S5: Oxidative Stress Related Pfam Domains

Supplementary Table S6: Genome statistics and abundances of 444 actinobacterial bins

Supplementary Table S7: TIGRFAMs markers used for phylogenomic analyses
